# Gastrointestinal Stromal Tumours treated before and after the advent of c-kit immunostaining

**DOI:** 10.1186/1477-7819-9-44

**Published:** 2011-04-27

**Authors:** Paolo G Sorelli, Patrizia Cohen, Bafour Amo-Takyi, Nikitas A Theodorou, Peter M Dawson

**Affiliations:** 1 Department of GI Surgery, Imperial College NHS Trust, Charing Cross Hospital, Fulham Palace Road, London, W6 8RF, UK; 2 Department of Histopathology, Imperial College NHS Trust, Charing Cross Hospital, Fulham Palace Road, London, W6 8RF, UK

## Abstract

**Background:**

Recently developed immunohistochemical markers have revolutionised the classification of gastrointestinal stromal tumours (GISTs) whilst tyrosine kinase inhibitors (imatinib) have had a significant impact on the treatment of advanced tumours. We review the clinicopathological features of previously resected mesenchymal tumours of the gastrointestinal tract in our institution to 1) reclassify the histological diagnosis of those stained prior to c-kit availability; 2) perform survival analysis to identify prognostic factors, and 3) to consider the implications for patients.

**Methods:**

Clinicopathological records of patients with a diagnosis of mesenchymal tumours treated between May 1992 and April 2007 were reviewed.

**Results:**

82 patients were reviewed. 26 (32%) were reclassified as GISTs following c-kit immunostaining and a further 14 patients were treated for GIST up to April 2007 (Total: 40 patients; 21 males and 19 females, mean age 67, range 30-92 years). 36 (90%) underwent complete resection. 5-year survival of patients with GIST alone was 80%. Females had a better median survival (M: F 43 months: 73 months).

**Conclusions:**

The availability of c-kit staining allowed 32% of previously diagnosed mesenchymal tumours to be reclassified as GISTs. This may have implications for the follow-up of patients diagnosed prior to the availability of this method.

## Introduction

Gastrointestinal stromal tumours (GISTs) are the most common form of mesenchymal (connective tissue) tumours of the GI tract. They are rare and represent approximately 0.3-3% of all gastrointestinal tumours [1]. In the last decade studies have discovered that nearly all GISTs are characterised by the expression of the c-kit receptor (CD117), as is their cell of origin, the interstitial cell of Cajal [2]. Detection of the c-kit protein in tumour cells by immunohistochemistry is now the standard criterion for the diagnosis of GIST. The most recent estimated incidence of GIST ranges between 1.1-1.46/100 000 per year based on national epidemiological studies [3-5].

Surgery is the standard of care for primary disease. However 40%-90% of surgically resected tumours recur [6]. Overall survival after surgical resection and clinical behaviour of GIST are dependent on tumour size and mitotic count [7,8]. All tumours have the potential to become malignant and Fletcher et al [9] proposed a scheme for defining the risk of aggressive behaviour of GIST into different classes, based on tumour size and mitotic count [10,11].

GISTs can occur anywhere in the GI tract, however most GISTs arise in the stomach or small intestine and infrequently in the colon or rectum, oesophagus, mesentery, or omentum, [12,13]. Patients may present with few or no symptoms depending on the size and location of the tumour mass. Small GISTs (2 cm or less) are usually asymptomatic and found incidentally during investigations or surgical procedures for unrelated causes. Larger GISTs can present with GI bleeding, a palpable mass, obstruction or abdominal pain.

The aim of our study was to review the clinicopathological features of previously resected mesenchymal tumours of the GI tract in our institution in order to 1) reclassify the histological diagnosis of those stained prior to c-kit availability, 2) perform survival analysis to identify prognostic factors, and 3) consider the implications of informing patients of their new diagnosis.

## Materials & methods

A retrospective review of casenotes of patients treated for mesenchymal tumours of the GI tract between May 1992 and April 2007 was performed. The patients' age, gender, tumour site and size after resection, date of surgery, extent of surgical resection, risk group according to the classification proposed by Fletcher et al, the presence and date of local recurrence or distant metastasis, and the clinical outcome until last follow-up, including date of death where appropriate, were recorded. Statistical analysis was performed using SPSS 12.0 software (Chicago, IL, USA). Overall actuarial survival was calculated from the day of surgery until death or the last day of a patient's visit to the outpatient clinic. Survival curves were plotted using Life Tables, and multivariate analysis performed using Cox regression analysis. Curves were compared using Lee-Desu statistics or Chi-Square.

Histopathological re-examination of surgical specimens was carried out by 2 Consultant Histopathologists (PC/BA) using standard hematoxylin and eosin staining as well as specific immunohistochemical techniques allowing the identification of tumour's size and number of mitosis at high-power field (HPF). Mutational analysis for c-KIT/PDGFRA tyrosine kinase receptor genes was not performed.

## Results

82 specimens previously classified as mesenchymal tumours of the GI tract between May 1992 and July 2003 were reviewed histologically of which 26 (32%) were reclassified as GIST following c-kit immunostaining. A further 14 patients were treated for GIST up to April 2007 following routine c-kit staining, introduced in our institution in 2003. A total of 40 patient casenotes were reviewed (21 males and 19 females, mean age 67, range 30-92 years). There was no significant difference in measured parameters between the patients identified retrospectively prior to routine c-kit immunostaining, and those identified after its routine adoption. At time of study 18 (45%) patients had died, 12 (30%) were lost to follow up.

Tumours were located in the stomach, small bowel, large bowel and retroperitoneum in 24, 7, 5 and 4 cases respectively. Using the "risk of aggressive behavior" classification proposed by Fletcher *et al *tumours were classified as very low (6/40), low (9/40), intermediate (6/40) and high-risk (19/40). Four patients had separate primary malignancies at presentation (rectal 1, stomach 2, sigmoid 1) with the GIST tumour resected as an incidental finding. 3 patients went on to develop separate primary tumours following complete resection of GIST (colorectal 1, ovary 1, bladder 1).

**Figure 1:**
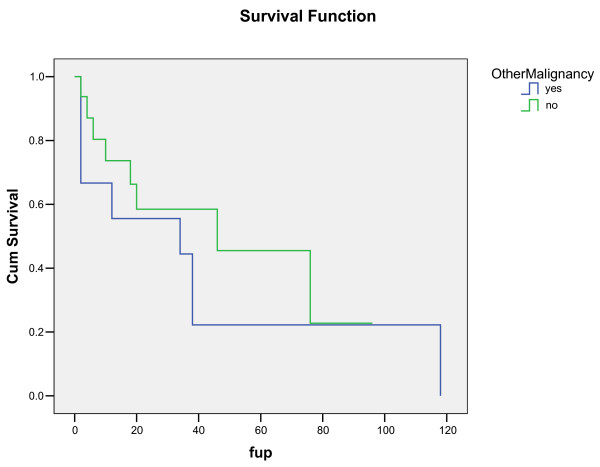
**Life Table showing survival by separate malignancy**.

Thirty-six patients (90%) underwent complete resection. Three (7.5%) had incomplete resection due to degree of local invasion, all of which were high risk GISTs. 1 patient had unresectable disease and underwent chemotherapy. The 5 year overall survival was 42% with a median survival of 46 months. The 5 year survival of patients with GIST alone irrespective of complete resection was 80%. The presence of separate malignancy significantly decreased 5 year survival from 50% to 13% (median survival from 100 months to 32 months) which reached statistical significance (p = 0.041) (Figure 1). Complete resection of GIST in these patients did not affect 5 year survival. Median survival after complete and incomplete resection for patients with GIST alone was 74 months versus 11 months respectively. High-risk tumours had a shorter survival than low and very low risk tumours (93 months versus 43 months). There was no statistically significant difference in survival by the location of tumour. Females had a better median survival than males both overall (median survival 73 months versus 43 months) and in the patients without separate malignancy (median survival 73 months versus 37 months). Multivariate analysis identified complete resection and sex as the most important positive prognostic factors, and the presence of separate malignancy as the most important negative prognostic factor for survival. Of the 26 patients who were reclassified as having GISTs, 16 have died and 10 were lost to follow up.

## Discussion

In this study we have demonstrated that a third of patients previously diagnosed with mesenchymal tumours of the gastrointestinal tract treated before the advent of c-kit immunostaining were reclassified as GISTs following positive immunohistochemical staining with c-kit.

Radical surgery to achieve complete resection remains the treatment of choice. The aim of treatment for GISTs identified with c-kit immunostaining, and tumours treated pre c-kit staining as mesenchymal tumours of the GI tract, remains complete resection. Therefore in our study management of the patients treated for GIST pre and post c-kit staining was the same. Previous studies have reported 5 year survival following complete resection between 35-75% [8,10,14-16]. Our findings suggest even higher 5 year survival rates can be achieved. Treatment may be improved by neoadjuvant or adjuvant imatinib therapy although its potential role for large or incompletely resected tumours is yet to be confirmed [17,18]. Imatinib has been shown to be effective in locally advanced and irresectable or metastatic GISTs [19,20] and its use is now recommended in these patients [21]. No patients in our study were eligible for imatinib therapy.

We found no significant correlation between site of GIST and survival. Miettinen et al [22] have suggested that gastric GISTs have a more favourable prognosis than intestinal ones with similar histological parameters. They proposed a different classification of GISTs into 8 subgroups in relation to size and mitotic rate in order to better define the rate of metastases in GISTs at different sites. Survival analysis in our population of GISTs according to Miettinen's classification are summarised in Table 1. In the less aggressive histological groups gastric GISTs had an overall improved median survival when compared to small bowel GISTs, however the improved survival was reversed in the more aggressive histological groups. More recently Tryggvason et al confirmed with their series that nongastric GISTs had a clearly higher risk of malignant behaviour than gastric GISTs [4].

**Table 1:** Median survival in GISTs of stomach and small intestine by tumours grouped by mitotic rate and tumour size as proposed by Miettinen et al^21 ^(n = 40)

	Tumour parameters		Median survival (months)		
**Group**	**Size**	**Mitotic rate**	**Gastric**	**Small bowel**	**Other**

1	≤2 cm	≤5 per 50 HPFs			
			34	2	60
2	>2≤5 cm	≤5 per 50 HPFs			

3a	>5≤10 cm	≤5 per 50 HPFs			
			44	-	53
3b	>10 cm	≤5 per 50 HPFs			

4	≤2 cm	>5 per 50 HPFs			
			-	-	-
5	>2≤5 cm	>5 per 50 HPFs			

6a	>5≤10 cm	>5 per 50 HPFs			
			44	60	36
6b	>10 cm	>5 per 50 HPFs			

We demonstrated a survival benefit in females in both univariate and multivariate analysis. Keun Park et al [23] have also recently found a survival advantage in females but only in univariate analysis. Comparison of the two groups in our study did not reveal any obvious confounding factors, however further analysis with larger numbers are required to reveal any statistical significance to this finding.

The optimal follow-up protocol for resected GISTs has not been firmly established. Recurrence appears to be possible even after many years and may be of slow onset. GISTs are resistant to conventional chemotherapy with a poor response rate (7-20%), and short response duration (1-4 months) [24]. Imatinib is effective therapy for patients with advanced metastatic or unresectable GIST and its use has significantly changed the management and prognosis for these patients [20,21]. Keun Park et al [23] showed that adjuvant imatinib for primary resected intermediate and high-risk GISTs significantly improved recurrence-free survival, and more recently DeMatteo et al [25] confirmed, following a randomised, double-blind, placebo-controlled trial, that imatinib improves recurrence-free survival following complete resection of GISTs.

Some clear recommendations of management of such retrospectively identified cases would be of value. The question arises as to whether patients previously diagnosed with mesenchymal tumours should now have their diagnosis reviewed with the aim of identifying patients who may be at risk of recurrence and therefore may benefit from follow up with a view to earlier diagnosis and further treatment. Given the time for which c-kit has now been available and in regular use it is likely that such patients have either succumbed to recurrence of the disease or are free and cured with little affect on their prognosis. However this data would be important to further elucidate this as well as the natural history and progression of such tumours, and the impact that c-kit has had on overall prognosis of patients suffering from GIST.

## Conclusions

The wide spectrum of histological terms that have been used in the past to identify GIST has made definition of its real incidence and survival difficult. Our study shows that approximately 1/3 of previously diagnosed mesenchymal tumours of the gastrointestinal tract could now be reclassified as GISTs. We confirm that the groups of risk of aggressive behaviour defined by Fletcher et al are significantly related to prognosis, with the low and very low risk category having improved median survival compared to the high risk group. Complete resection remains the most important prognostic factor and we have demonstrated that an 80% 5 year survival can be achieved in our cohort of patients. The advent of new therapies may have implications for further possible treatment in patients with advanced or recurrent disease. This therefore raises the question as to whether the diagnosis of all previously undefined mesenchymal tumours of the GI tract where c-kit immunostaining was not performed should be revised and patients with confirmed GISTs contacted with a view to further follow up.

## Competing interests

The authors declare that they have no competing interests.

## Authors' contributions

PS, NT and PD guarantee the integrity of the entire study. PS, PC, NT and PD developed the study concepts and design. PS undertook the literature research. BA and PC performed the histological studies. PS and PD performed the data and statistical analysis. PS prepared the manuscript. All authors read and approved the final manuscript.
